# Neuroprotective effects of naringenin on nicotine-induced anxiety and depression: Involvement of monoaminergic systems, oxidative stress, and neuroinflammation on male rats

**DOI:** 10.1016/j.crphar.2025.100242

**Published:** 2025-12-04

**Authors:** Murtaza Haidary, Yahya Samadi, Zakaria Rezai, Atiqullah Sadaqat, Mohammad Ali Ahmadi, Jamshid Gholami, Mohammad Mahdi Mohammadi, Mohammad Taqi Shojae

**Affiliations:** a Medical Research and Technology Center, Khatam Al-Nabieen University, Kabul, Afghanistan; b Department of Biology and Microbiology, Faculty of Medical Laboratory Technology, Khatam Al- Nabieen University, Kabul, Afghanistan

**Keywords:** Nicotine withdrawal, Adolescence, Serotonin, Dopamine, Oxidative stress, Neuroinflammation, Prefrontal cortex, Naringenin

## Abstract

**Introduction:**

Nicotine withdrawal during adolescence induces severe neurobehavioral disturbances and neurochemical alterations, including anxiety, depression, affective dysregulation, oxidative stress, and neuroinflammation. Current therapeutic options for managing nicotine dependence remain suboptimal. This study investigated the neuroprotective potential of naringenin (NG) in alleviating behavioral and biochemical sequelae of nicotine withdrawal in adolescent rats.

**Materials and methods:**

Male adolescent Wistar rats were allocated into eight groups and subjected to nicotine exposure (1 mg/kg) and NG treatment (50 or 100 mg/kg) across nicotine exposure and withdrawal phases. Behavioral assays (OFT, EPM, FST) were employed to evaluate anxiety- and depression-like behaviors. Neurochemical assessments of dopamine, serotonin, their metabolites (DOPAC, 5-HIAA), MAO-A activity, oxidative stress markers (MDA, Nit), antioxidant enzymes (SOD, CAT, TT), and neuroinflammatory/neurodegenerative biomarkers (GFAP, IL-10, BDNF, NSE) were conducted in prefrontal cortex (PFC) homogenates.

**Results:**

Nicotine withdrawal significantly induced anxiety- and depression-like behaviors, disrupted monoaminergic balance, elevated MAO-A activity, and triggered oxidative and neuroinflammatory responses in the PFC. NG administration, particularly at 100 mg/kg across both phases, significantly ameliorated behavioral impairments, restored neurotransmitter homeostasis, inhibited MAO-A, suppressed lipid peroxidation and nitrosative stress, enhanced antioxidant defenses, reduced GFAP and NSE expression, and restored IL-10 and BDNF levels.

**Conclusion:**

NG exerts anxiolytic, antidepressant, antioxidant, and anti-inflammatory effects, likely via modulation of monoaminergic pathways and suppression of neuroinflammation and oxidative stress. These findings underscore the potential of NG as a promising candidate for mitigating neuropathological effects associated with nicotine withdrawal-induced neuropathology, particularly during adolescence.

## Highlights

• Adolescent nicotine withdrawal induced profound neurobehavioral impairments, monoaminergic dysregulation, and oxidative–inflammatory damage in the prefrontal cortex.
• Naringenin treatment alleviated anxiety- and depression-like behaviors associated with nicotine withdrawal in adolescent rats.
• Naringenin restored serotonergic and dopaminergic homeostasis and suppressed MAO-A hyperactivity induced by nicotine exposure.
• Naringenin reduced oxidative stress and neuroinflammation by lowering MDA, nitric oxide, GFAP, and NSE, and enhancing SOD, CAT, IL-10, and BDNF.
• These findings support naringenin as a multi-target neuroprotective agent against nicotine-induced neuropathology during adolescence.

## Graphical abstract

**Figure:**
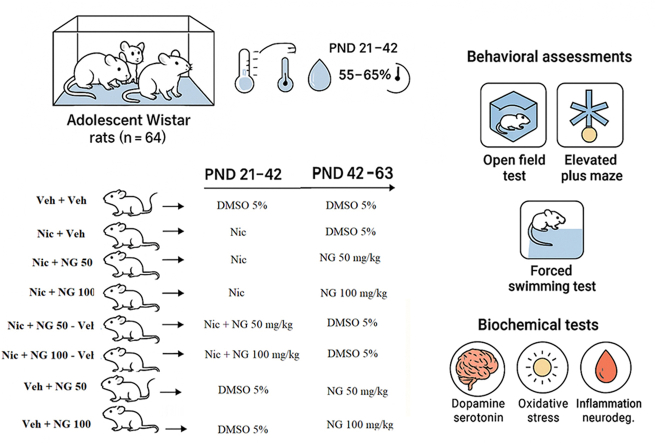

## 1 Introduction

Adolescence represents a vulnerable period in which nicotine use poses serious public health challenges, largely due to the increased susceptibility to addiction and the enduring consequences of nicotine exposure during this critical stage of neurodevelopment (Hossaini et al., 2025a). This phase is marked by dynamic structural and functional changes in the central nervous system (Baum et al., 2022), including substantial remodeling of neurotransmitter systems, particularly those governing reward and motivation (Bonke et al., 2023). A key neurochemical action of nicotine is its ability to elevate dopamine levels within the brain's mesolimbic reward circuitry, reinforcing its addictive potential (Subramaniyan and Dani, 2015; Markou, 2008). Additionally, nicotine stimulates the release of serotonin, a neurotransmitter integral to mood regulation and cognitive function (D'Souza and Markou, 2013; Al-Wadei et al., 2009).

Nicotine's acute effects may include transient improvements in attention and memory due to its modulation of neurotransmitter systems. However, with chronic exposure, these initial enhancements often give way to cognitive deficits and withdrawal symptoms upon cessation (JARVIK, 1991; Marcela Waisman et al., 2016). Withdrawal is typically marked by a decline in central dopamine (Pandey et al., 2001) and serotonin levels, which can precipitate adverse emotional states such as anxiety and depression (Hossaini et al., 2025b). These neuropsychological symptoms are attributed to dysregulation of dopaminergic and serotonergic pathways, underscoring the biological complexity of nicotine dependence and cessation difficulties (Pandey et al., 2001). Long-term nicotine exposure has been shown to induce persistent alterations in neurotransmitter signaling, thereby heightening the risk of developing mood and anxiety disorders even after cessation (Jobson et al., 2018; Alhusban et al., 2023). Moreover, chronic nicotine intake elevates oxidative stress levels, compromising the blood-brain barrier (BBB) and initiating neuroinflammatory responses. This oxidative damage is associated with microglial activation and the release of pro-inflammatory cytokines, processes that are implicated in neurodegeneration and neuropsychiatric conditions (Leszek et al., 2016; Sajja et al., 2016). As such, nicotine use is closely linked to the pathophysiology of anxiety, depression, neuroinflammation, and imbalances in neurotransmitter function (Paz et al., 2007; Amiry et al., 2023; Ahmadi-Soleimani et al., 2023).

Although current pharmacological therapies are available to manage nicotine withdrawal, ongoing research is exploring alternative interventions that are both safer and more effective (Yammine et al., 2021). Naringenin (NG) is a naturally occurring flavanone primarily present in citrus fruits such as oranges, mandarins, grapefruits, and lemons, as well as in some vegetables. A diet rich in NG-containing fruits and vegetables has been linked to a reduced risk and improved management of several chronic diseases, including obesity, hypertension, cardiovascular disorders, metabolic syndrome, and neurodegenerative conditions (Alam et al., 2014). Pharmacokinetic studies have shown that NG can efficiently cross the BBB and is rapidly metabolized in the liver into glucuronide derivatives (Wang et al., 2006). This high BBB permeability is believed to underlie NG's broad spectrum of central nervous system (CNS) effects (Youdim et al., 2004). An increasing body of literature supports NG's neuroactive properties, underscoring its potential in the prevention and treatment of various neurological disorders (Khan et al., 2012; Raza et al., 2013; Ghofrani et al., 2015). Moreover, NG has been extensively reported to possess strong anti-inflammatory and antioxidant capacities (Alam et al., 2014; Chtourou et al., 2014; Cavia‐Saiz et al., 2010). However, despite these well-documented bioactivities, limited experimental data are available on the neuroprotective role of NG in nicotine-induced neurochemical disturbances. Therefore, the present study is designed to explore the impact of NG on nicotine-induced impairments in serotonin and dopamine metabolism, with particular emphasis on its role in attenuating oxidative stress and neuroinflammation in the prefrontal cortex (PFC).

## 2 Materials and Methods

### 2.1 Animals

Sixty-four adolescents male Wistar rats were obtained from the animal facility of Khatam Al-Nabieen University at postnatal day 21 (PND 21) and housed in groups of three to four per open-top plexiglass cage. The rats were kept under standardized environmental conditions, featuring a controlled temperature of 22 ± 2 °C, humidity levels maintained between 55 % and 65 %, and a 12-h light-dark cycle, with lights turning on at 6:00 a.m. The animals received a standard laboratory diet (Javaneh Khorasan, Mashhad, Iran) containing 46 % nanofibrillated cellulose, 25 % neutral detergent fibers, 19 % protein, and 10 % lipids, with ad libitum access to clean water. All experimental procedures were approved by the Animal Ethics Committee of the author's university (AF, knu.edu.af.rec 23, 1/5/2025) and were conducted in strict accordance with the Guide for the Care and Use of Laboratory Animals (Ahmadi-Noorbakhsh et al., 2021).

### 2.2 Experimental design and drug administration

**Fig. 1:**
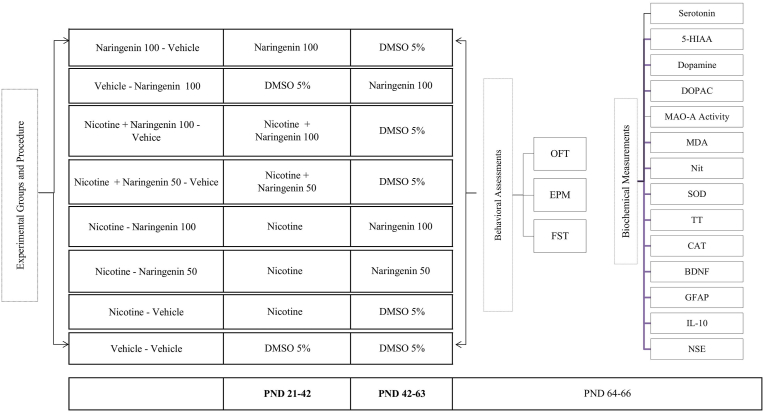
*A detailed timeline outlining the key phases and experimental events in the study.***Abbreviations:** PND: Postnatal Day; NG: Naringenin; OFT: Open Field Test; EPM: Elevated Plus Maze; FST: Forced Swim Test; PFC: Prefrontal Cortex; DOPAC: 3,4 Dihydroxyphenylacetic Acid; (5-Hydroxytryptamine); 5-HIAA: 5 Hydroxyindoleacetic Acid; MAO-A: Monoamine Oxidase A; MDA: Malondialdehyde; SOD: Superoxide Dismutase; CAT: Catalase; TT: Total Thiol; Nit: Nitrite; GFAP: Glial Fibrillary Acidic Protein; BDNF: Brain-Derived Neurotrophic Factor; IL-10: Interleukin-10; NSE: Neuron-Specific Enolase.

(−)-Nicotine base (MFCD00006369) and NG (W530098) were procured from Sigma-Aldrich. Nicotine was diluted in normal saline (0.9 % sodium chloride), whereas NG was initially dissolved in 5 % dimethyl sulfoxide (DMSO). The 5 % DMSO solution also served as the vehicle control for NG administration. NG was administered intraperitoneally (i.p.) at doses of 50 and 100 mg/kg, selected based on previous studies demonstrating their pharmacological efficacy (Olugbemide et al., 2021). Nicotine was administered subcutaneously (s.c.) at a dose of 1 mg/kg, twice daily at 6:00 a.m. and 6:00 p.m. This dosage has been shown in previous studies to be non-toxic (Akimoto et al., 2018; Kota et al., 2008). The experimental design included eight groups of adolescent rats (n = 8), treated across PND 21–42, corresponding to the nicotine exposure period, and PND 42–63, representing the post-exposure (withdrawal) phase. The sample size per group (n = 8) was determined based on previous studies using rodent models of nicotine dependence and withdrawal, which employed similar behavioral and neurochemical endpoints and demonstrated that n = 8 per group was sufficient to detect significant treatment effects with acceptable variability (Malin et al., 1992; Muelken et al., 2015). Group allocations were as follows: Group 1 (Vehicle – Vehicle): Received 5 % DMSO throughout both phases (PND 21–63) and served as the negative control. Group 2 (Nicotine – Vehicle): Administered nicotine (1 mg/kg, s.c.) twice daily during the exposure phase (PND 21–42), followed by 5 % DMSO during the post-exposure period (PND 42–63), to assess the effects of nicotine and subsequent withdrawal. Groups 3 and 4 (Nicotine – NG): Received nicotine the same like group 2 during PND 21–42, followed by NG at 50 mg/kg (Group 3) or 100 mg/kg (Group 4), i.p., during PND 42–63. These groups were designed to evaluate the efficacy of NG as a post-withdrawal therapeutic intervention. Groups 5 and 6 (Nicotine + NG – Vehicle): Co-administered nicotine like the same group 2 and NG (50 mg/kg for Group 5 and 100 mg/kg for Group 6, i.p.) during PND 21–42, followed by 5 % DMSO during PND 42–63. These groups assessed the neuroprotective potential of NG during active nicotine exposure. Group 7 (NG – Vehicle): Received NG alone (100 mg/kg, i.p.) from PND 21 to 42, followed by 5 % DMSO during PND 42–63. This group evaluated the standalone effects of NG during the exposure window. Group 8 (Vehicle – NG): Treated with 5 % DMSO during PND 21–42, followed by NG (100 mg/kg, i.p.) during PND 42–63, to assess the therapeutic potential of delayed NG administration following nicotine exposure. A schematic representation of the experimental timeline and treatment regimens is provided in Fig. 1.

### 2.3 Behavioral assessment protocol

It has been shown that during the first week following nicotine cessation, adolescent rats typically do not exhibit pronounced anxiety-like behaviors (Chellian et al., 2021). Therefore, behavioral testing commenced on postnatal day 64, 21 days after the initiation of the withdrawal period, to allow stabilization of withdrawal-related symptoms. All assessments were conducted during the light phase of the 12:12 h light/dark cycle (between 09:00 and 14:00 h) to ensure consistency and minimize circadian variability. The test sequence was arranged from less stressful to more stressful paradigms, beginning with the open field test (OPT) to evaluate locomotor and exploratory activity, followed by the elevated plus maze (EMP) for anxiety-like behavior, and concluding with the forced swim test (FST) for depressive-like behavior. This time point was strategically chosen to capture the intermediate phase of withdrawal, during which emotional and cognitive disturbances are more likely to emerge. To minimize stress-related variability and enhance the reliability of behavioral data, all animals were acclimatized to the testing room for a minimum of 30 min prior to each test. Lighting conditions were standardized based on task-specific requirements: 150 lux for the EPM to promote open-arm exploration and 40 lux for the OFT to allow natural exploratory behavior without artificially elevating anxiety levels. To prevent potential confounding effects, animals that completed behavioral testing were housed separately from those awaiting evaluation. In addition, all apparatuses were thoroughly cleaned between trials using a 10 % ethanol solution to eliminate residual olfactory cues from previous subjects, thereby ensuring consistency and validity across behavioral measurements.

#### 2.3.1 Open field test

The OFT is a well-established behavioral paradigm widely used to assess anxiety-like behavior and to investigate the neurobiological mechanisms underlying anxiety. It is also commonly employed to evaluate the anxiolytic potential of pharmacological agents (Kraeuter et al., 2019a). In this assay, animals are placed in a large, novel arena where their spontaneous locomotor activity and exploration patterns are observed. The test is based on the natural conflict between the animal's aversion to open, exposed areas and its innate drive to explore new environments. One of the most frequently measured indicators of anxiety is the time spent in the central zone of the arena; increased time in the center is typically interpreted as a reduction in anxiety-like behavior (Nosek et al., 2008; Gould, 2009; Sturman et al., 2018). In the present study, the OFT was conducted to assess anxiety-related behaviors in adolescent rats. The testing apparatus consisted of a square arena measuring 100 × 100 × 40 cm, constructed from opaque, non-reflective material to minimize external visual distractions and reflections. Each rat was gently placed in the center of the arena and allowed to explore freely for 5 min. This duration was selected as it provides sufficient time to capture reliable behavioral data while minimizing habituation effects, which could otherwise alter the animal's response to the novel environment (Delprato et al., 2017). Additionally, this time frame is optimal for detecting initial anxiety responses, which are critical for evaluating the effects of pharmacological interventions or experimental manipulations (Mesiakaris et al., 2024). Throughout the testing session the time spent in the central versus peripheral zones—were recorded using a video tracking system.

#### 2.3.2 Elevated plus maze

Following the OFT, the anxiolytic effects of NG were further evaluated using the EPM—a widely accepted behavioral assay for assessing anxiety-like behavior and general locomotor activity in rodents. The EPM apparatus consists of a plus-shaped platform elevated 50 cm above the ground, comprising two open arms and two enclosed arms (with 40 cm high walls) arranged perpendicularly around a central square. This setup exploits the animal's natural conflict between the fear of open, elevated areas and the innate drive to explore novel environments (Acikgoz et al., 2022). In this study, the maze was constructed from opaque gray wood to reduce external visual cues and promote a controlled testing environment. Each rat was placed in the central zone of the maze, facing one of the open arms, under dim lighting conditions conducive to exploration without increasing stress levels. Animals were then allowed to explore freely for 5 min, and their behavior was recorded using an automated tracking system. The primary behavioral metrics analyzed included the time spent in the open arms versus the closed arms, which served as indices of anxiety-like behavior. A greater proportion of time spent in the open arms is typically interpreted as reduced anxiety, whereas a preference for the closed arms indicates elevated anxiety levels.

#### 2.3.3 Forced swimming test

The FST is a widely used behavioral paradigm for evaluating depression-like behavior in rodents. In this test, animals are placed in a water-filled container from which they cannot escape, and their behavioral responses are monitored to assess emotional states such as behavioral despair. The duration of immobility—defined as the absence of active escape-directed behaviors—is considered an index of depressive-like behavior and is often used to evaluate the efficacy of antidepressant treatments (Pumpaisalchai et al., 2005). In the present study, the FST was employed to assess depression-like behavior in adolescent rats. Each rat was individually placed in a transparent glass cylinder (height: 50 cm; diameter: 20 cm) filled with water to a depth of 30 cm, maintained at a temperature of 24 ± 2 °C. This water depth was sufficient to prevent the animals from touching the bottom, thereby requiring continuous effort to stay afloat. The total test duration was 5 min, during which each animal was gently placed into the water, and its behavior was continuously recorded using a video camera for subsequent analysis. Behavioral responses were classified into three distinct categories. Struggling was defined as vigorous, coordinated movements involving both the forelimbs and hind limbs, typically accompanied by attempts to climb the walls of the cylinder. Swimming referred to active horizontal movement across the surface of the water without any climbing behavior. In contrast, immobility was characterized by the absence of active movement, with only minimal motions necessary to keep the animal's head above water, indicating a state of behavioral despair.

### 2.4 Euthanasia

Following the behavioral assessments, animals were euthanized using a gradual-fill carbon dioxide (CO_2_) method with a 95 % CO_2_ gas mixture. CO_2_ was introduced into the euthanasia chamber at a controlled rate of 50 % of the chamber's volume per minute (Hossaini et al., 2025b), in accordance with the American Veterinary Medical Association (AVMA) Guidelines for the Euthanasia of Animals: 2020 Edition, which recommend a displacement rate of 30–70 % to minimize animal distress and avoid aversive responses associated with rapid gas exposure (Underwood and Anthony, 2020). Euthanasia was confirmed based on the cessation of respiratory movements, absence of a detectable heartbeat (via thoracic palpation), and loss of reflexes, including the pedal withdrawal reflex. Only after all signs of life were absent and unresponsiveness was verified were animals considered deceased. Immediately after death was confirmed, brains were carefully extracted. The PFC was dissected with precision and rinsed with 0.9 % sterile normal saline to remove residual blood and prepare the tissue for biochemical assays. To perform the biochemical analyses, a 50 % (w/v) PFC tissue homogenate was prepared by homogenizing the PFC samples in 0.1 M phosphate buffer (pH 7.4). The homogenates were then centrifuged at 4 °C for 20 min to separate the supernatant, which was used for subsequent biochemical measurements.

### 2.5 Biochemical measurements

#### 2.5.1 Measurement of serotonin and dopamine metabolism

To evaluate changes in monoaminergic neurotransmission, the concentrations of dopamine, serotonin, and their primary metabolites— 3,4-Dihydroxyphenylacetic Acid (DOPAC) and 5-Hydroxyindoleacetic Acid (5-HIAA)—were quantified in PFC homogenates using enzyme-linked immunosorbent assay (ELISA) kits from MyBioSource, USA. The kits utilized included dopamine (Cat. No. MBS725908), DOPAC (MBS7269503), serotonin (MBS1608278), 5-HIAA (MBS700811), and Monoamine Oxidase A)MAO-A(activity (MBS721413). All assays were performed according to the manufacturers’ protocols. In brief, homogenate samples were added to microplate wells pre-coated with analyte-specific antibodies, followed by incubation with enzyme-conjugated detection antibodies. A chromogenic substrate was then applied, and the resulting colorimetric reaction—proportional to analyte concentration—was quantified by measuring absorbance with an ultraviolet–visible (UV–Vis) spectrophotometer (UV-1600, Shimadzu, Japan).

#### 2.5.2 Measurement of oxidative stress and antioxidant markers

Lipid peroxidation was assessed by measuring Malondialdehyde (MDA) levels using the thiobarbituric acid reactive substances (TBARS) assay. Briefly, 0.5 mL of tissue supernatant was mixed with 2.5 mL of 20 % trichloroacetic acid (TCA) and 1 mL of 0.67 % thiobarbituric acid (TBA) solution (Sigma-Aldrich, St. Louis, MO, USA). The mixture was heated at 95 °C for 30 min, cooled, and centrifuged at approximately 1500×*g* for 10 min. Absorbance was measured at 532 nm, and MDA concentration was calculated and expressed as nmol/mg tissue (Ohkawa et al., 1979; Akbari et al., 2023). Nitrate content, an indicator of nitric oxide metabolism and nitrosative stress, was measured using a commercial Nitrate (Nit) ELISA kit (Cat. No. MBS9718972, MyBioSource, USA) following the manufacturer's guidelines. Total Thiol (TT) groups were quantified employing Ellman's reagent (5,5′-dithiobis-(2-nitrobenzoic acid), DTNB; Sigma-Aldrich). For this assay, 50 μL of homogenate was combined with 1 mL of Tris-EDTA buffer (pH 8.6) and 50 μL of 10 mM DTNB, incubated at room temperature for 15 min, and absorbance recorded at 412 nm. Thiol concentrations were calculated using a molar extinction coefficient of 13,600 M^-1^cm^-1^ and expressed as μmol/mg protein (Ellman, 1959).

Superoxide Dismutase (SOD) activity was determined using a commercial colorimetric kit (e.g., Cayman Chemical, Cat# 706002) based on the inhibition of a tetrazolium salt (WST-1) reduction by superoxide radicals. The formation of a colored formazan product was measured at 450 nm, with one unit of SOD defined as the enzyme amount causing 50 % inhibition of color development. SOD activity was calculated via comparison with a standard curve provided by the kit.

Catalase (CAT) activity was measured spectrophotometrically by monitoring the decomposition rate of hydrogen peroxide (H_2_O_2_) according to Aebi's method (1984). In this assay, 50 μL of tissue homogenate was mixed with 2.95 mL of 30 mM H_2_O_2_ prepared in 50 mM phosphate buffer (pH 7.0), and the decrease in absorbance at 240 nm was recorded over 1 min. Results were expressed as μmol H_2_O_2_ decomposed per minute per mg protein (Akbari et al., 2023; Aebi, 1984).

### 2.6 Measurement of neuroinflammatory and Neurodegenerative Markers

To assess neuroinflammatory responses and neurodegeneration in PFC, levels of key protein markers were measured using commercially available ELISA kits. Glial fibrillary acidic protein (GFAP) was quantified using the GFAP ELISA kit (Cat. No. MBS042687, MyBioSource, USA). IL-10 was measured using the interleukin-10 (IL-10) ELISA kit (Cat. No. MBS2021530, MyBioSource, USA). To evaluate neuronal injury, levels of neuron-specific enolase (NSE) were determined using the NSE ELISA kit (Cat. No. MBS729794, MyBioSource, USA). In addition, brain-derived neurotrophic factor (BDNF) was quantified using a specific BDNF ELISA kit from CUSABIO (Cat. No. CSB-E04504r, USA). All assays were performed according to the respective manufacturers’ protocols. Absorbance readings were acquired using a UV–Vis spectrophotometer (Model: UV-1600, Shimadzu, Japan) at appropriate wavelengths, and concentrations were calculated based on standard curves provided in the kits.

### 2.7 Statistical analyses

All data were analyzed using GraphPad Prism software (version 8.4.3, GraphPad Software, San Diego, CA, USA). Statistical comparisons among groups were performed using one-way analysis of variance (ANOVA), followed by Tukey's post hoc test to assess pairwise differences. Results are expressed as mean ± standard error of the mean (SEM). A p-value of less than 0.05 (p < 0.05) was considered statistically significant, corresponding to a 95 % confidence level.

## 3 Results

### 3.1 NG mitigates anxiety-like behaviors associated with nicotine withdrawal

**Fig. 2:**
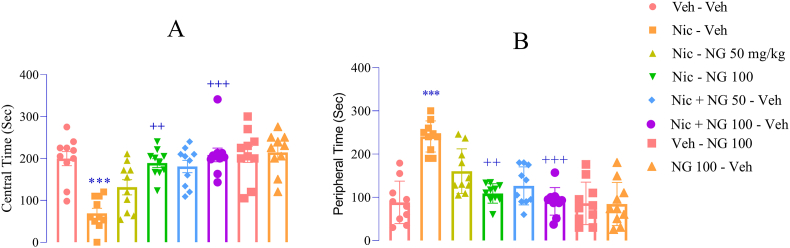
*Effects of Naringenin on Anxiety-Related Behaviors in the Open Field Test (OFT).* (A) Time spent in the central zone; (B) Time spent in the peripheral zone. Data are shown as mean ± SEM (n = 8). Significant differences: *p* < 0.05, ∗∗*p* < 0.001 vs. vehicle group; +*p* < 0.05, ++*p* < 0.01, +++*p* < 0.001 vs. nicotine group.

Behavioral analyses using the OFT revealed that nicotine withdrawal elicited pronounced anxiety-like responses in adolescent rats. Specifically, rats undergoing withdrawal spent significantly less time in the central zone of the arena (F (Al-Wadei et al., 2009; Wang et al., 2019) = 10.47, *p* < 0.001; Fig. 2A) and more time in the peripheral zones (F (Al-Wadei et al., 2009; Wang et al., 2019) = 10.95, *p* < 0.001; Fig. 2B), compared to the vehicle group. Notably, administration of NG at 100 mg/kg throughout both the nicotine exposure and withdrawal significantly increased central zone exploration (*p* < 0.01 and *p* < 0.001; Fig. 2A) and reduced peripheral zone occupancy (p < 0.01 and p < 0.001; Fig. 2B), compared to the nicotine group. In contrast, NG administered alone (100 mg/kg, without nicotine) had no significant effect on center or peripheral zone exploration compared to the vehicle group (Fig. 2A and B).

**Fig. 3:**
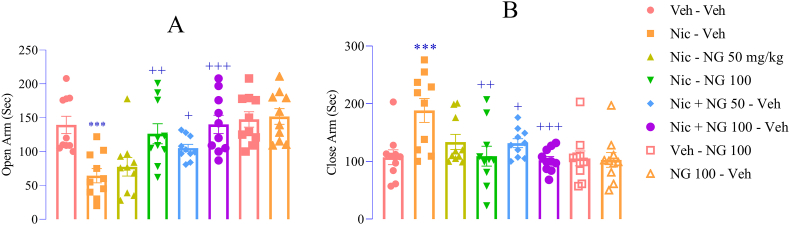
*Effects of Naringenin on Anxiety-Related Behaviors in the Elevated Plus Maze (EPM)* (A). Time spent in the open arms; (B) Time spent in the closed arms. Data are presented as mean ± SEM (n = 8). Significant differences: *p* < 0.05, ∗*p* < 0.01, ∗∗*p* < 0.001 vs. vehicle group; +*p* < 0.05, ++*p* < 0.01, +++*p* < 0.001 vs. nicotine group.

These observations were corroborated by the EPM test. Nicotine-withdrawn rats exhibited a marked reduction in time spent in the open arms (F (Al-Wadei et al., 2009; Wang et al., 2019) = 7.60, *p* < 0.001; Fig. 3A) and an increase in closed-arm exploration (F (Al-Wadei et al., 2009; Wang et al., 2019) = 4.64, *p* < 0.001; Fig. 3B) compared to the vehicle group. NG administration at 50 mg/kg during the nicotine withdrawal significantly increased open-arm time (*p* < 0.05; Fig. 3A) and reduced time spent in the closed arms (p < 0.05; Fig. 3B) compared to the nicotine group. Furthermore, NG at 100 mg/kg administered across nicotine exposure and withdrawal significantly increased in time spent in the open arms and reduced the time spent in the closed arms compared to the nicotine group (*p* < 0.01 and *p* < 0.001; Fig. 3A and B). However, NG given alone (100 mg/kg) did not significantly influence arm preference compared to vehicle group (Fig. 3A and B).

### 3.2 NG mitigates depression-like behaviors associated with nicotine withdrawal

**Fig. 4:**
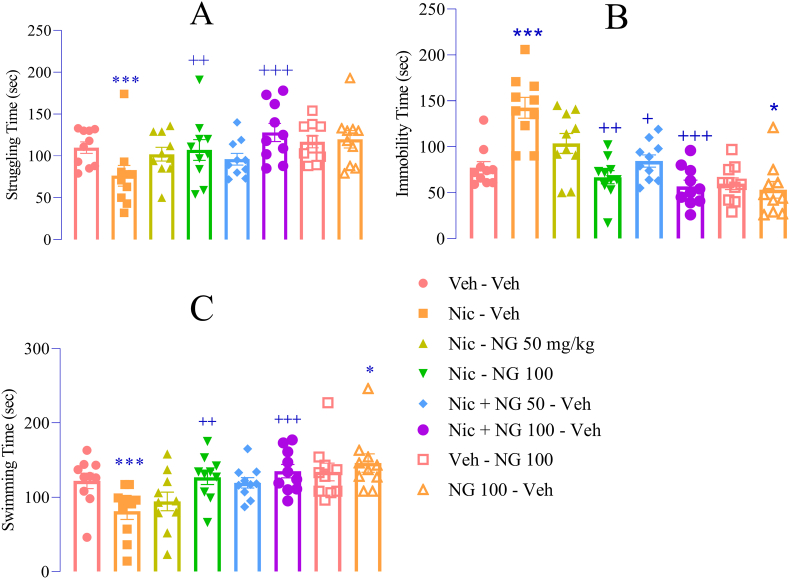
Effects of Naringenin on Depression-Like Behaviors in the Forced Swim Test (FST). (A) Struggling time; (B) Immobility time; (C) Swimming time. Data are shown as mean ± SEM (n = 8). Significant differences: p < 0.05, ∗p < 0.01, ∗∗p < 0.001 vs. vehicle group; +p < 0.05, ++p < 0.01, +++p < 0.001 vs. nicotine group.

In the FST, nicotine withdrawal significantly increased depressive-like behaviors, as evidenced by a reduction in struggling time (F (Al-Wadei et al., 2009; Wang et al., 2019) = 2.75, *p* < 0.001; Fig. 4A), an increase in immobility (F (Al-Wadei et al., 2009; Wang et al., 2019) = 13.02, *p* < 0.001; Fig. 4B), and a decrease in swimming time (F (Al-Wadei et al., 2009; Wang et al., 2019) = 4.23, *p* < 0.001; Fig. 4C) compared to the vehicle group. NG administration at 50 mg/kg during nicotine withdrawal significantly reduced immobility time (*p* < 0.05; Fig. 4B). Additionally, NG at 100 mg/kg, both during nicotine exposure and withdrawal, significantly increased struggling time (*p* < 0.01 and *p* < 0.001, respectively; Fig. 4A) and swimming time (p < 0.01 and *p* < 0.001, respectively; Fig. 4C), while reducing immobility (*p* < 0.01 and *p* < 0.001, respectively; Fig. 4B) compared to the nicotine group. Notably, NG at 100 mg/kg alone reduced immobility time and increased swimming time compared to the vehicle group (*p* < 0.05; Fig. 4B and C).

### 3.3 NG restores monoaminergic balance disrupted by nicotine withdrawal

**Fig. 5:**
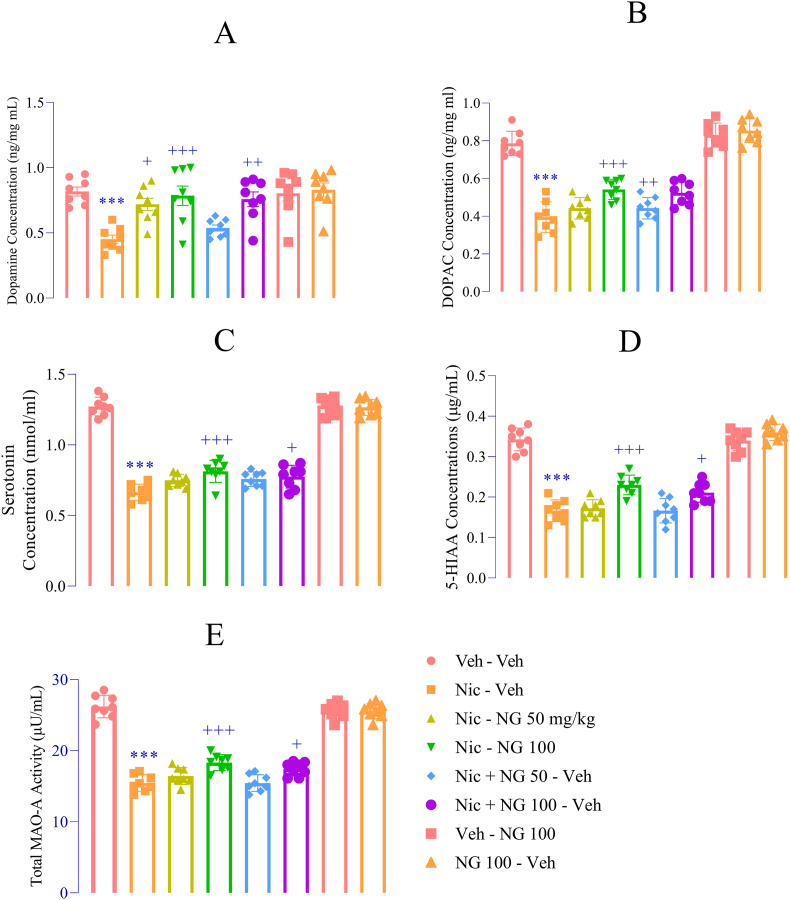
*Effects of Naringenin on Monoaminergic Transmission in the Prefrontal Cortex* (A) Dopamine levels; (B) DOPAC levels; (C) Serotonin levels; (D) 5-HIAA levels; (E) MAO-A activity. activity. Data are presented as mean ± SEM (n = 8). Significant differences: *p* < 0.05, ∗*p* < 0.01, ∗∗*p* < 0.001 vs. vehicle group; +*p* < 0.05, ++*p* < 0.01, +++*p* < 0.001 vs. nicotine group.

Nicotine withdrawal resulted in a significant reduction in dopamine concentrations compared to the vehicle control group (F (Al-Wadei et al., 2009; Wang et al., 2019) = 7.90, *p* < 0.001; Fig. 5A). In addition, levels of the dopamine metabolite DOPAC were significantly decreased (F (Al-Wadei et al., 2009; Wang et al., 2019) = 2.75, *p* < 0.001; Fig. 5B), along with marked reductions in serotonin (F (Al-Wadei et al., 2009; Wang et al., 2019) = 153.6, p < 0.001; Fig. 5C) and its primary metabolite 5-HIAA (F (Al-Wadei et al., 2009; Wang et al., 2019) = 91.20, p < 0.001; Fig. 5D). Conversely, MAO-A activity was significantly elevated in the nicotine-withdrawn group relative to vehicle (F (Al-Wadei et al., 2009; Wang et al., 2019) = 135.2, *p* < 0.001; Fig. 5E). Administration of NG at a dose of 50 mg/kg during nicotine exposure significantly elevated dopamine levels compared to the nicotine group (*p* < 0.05; Fig. 5A). A higher dose of 100 mg/kg, administered across nicotine exposure and withdrawal, produced a more pronounced increase in dopamine concentrations (*p* < 0.01 and *p* < 0.001, respectively; Fig. 5A). This dopaminergic enhancement was accompanied by significant elevations in DOPAC levels (*p* < 0.01 and *p* < 0.001, respectively; Fig. 5B). In parallel, NG at 100 mg/kg effectively restored serotonin levels during nicotine exposure and withdrawal (*p* < 0.05 and *p* < 0.001, respectively; Fig. 5C). This effect was corroborated by parallel increases in 5-HIAA levels (*p* < 0.05 and *p* < 0.001, respectively; Fig. 5D). Moreover, NG significantly suppressed nicotine withdrawal-induced upregulation of MAO-A activity (*p* < 0.05 and *p* < 0.001; Fig. 5E). Notably, NG administered alone—without nicotine exposure—did not produce any significant changes in dopamine, DOPAC, serotonin, 5-HIAA, or MAO-A activity compared to the vehicle group (Fig. 5A–E).

### 3.4 NG ameliorates oxidative stress in the prefrontal cortex

**Fig. 6:**
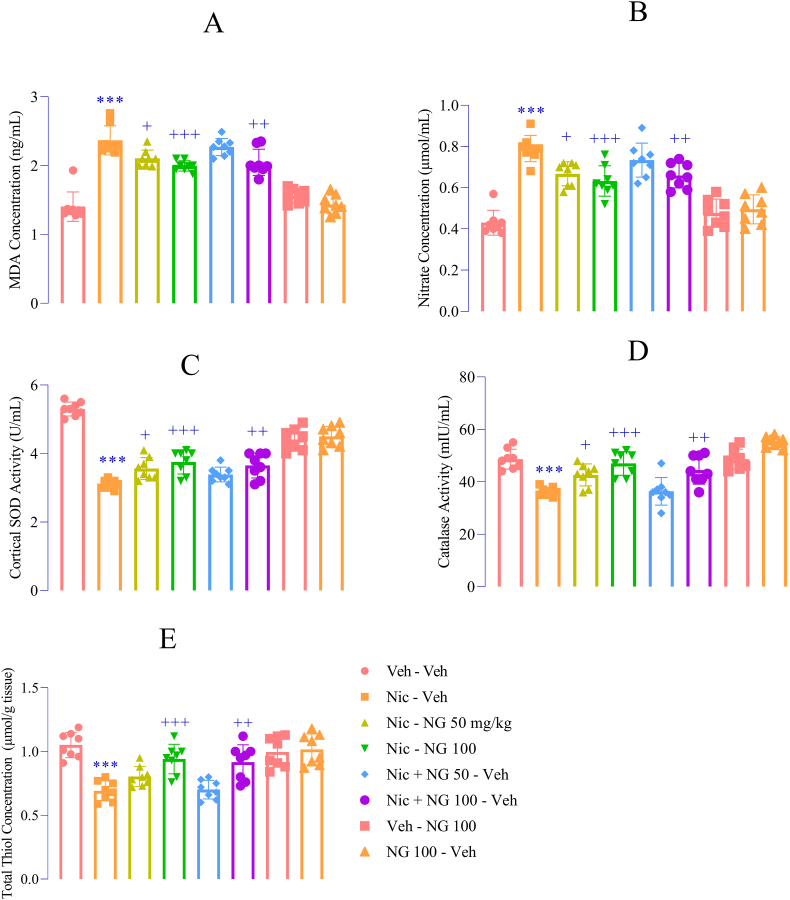
*Effects of Naringenin on Oxidative Stress Markers in the Prefrontal Cortex* (A) MDA levels; (B) Nitrite levels; (C) SOD activity; (D) CAT activity; (E) Total thiol (TT) levels. Data are shown as mean ± SEM (n = 8). Significant differences: *p* < 0.05, ∗*p* < 0.01, ∗∗*p* < 0.001 vs. vehicle group; +*p* < 0.05, ++*p* < 0.01, +++*p* < 0.001 vs. nicotine group.

Nicotine withdrawal significantly disrupted oxidative balance in the PFC. This was reflected by increased levels of lipid peroxidation marker MDA (F (Al-Wadei et al., 2009; Wang et al., 2019) = 47.79, *p* < 0.001; Fig. 6A) and Nit (F (Al-Wadei et al., 2009; Wang et al., 2019) = 29.42, *p* < 0.001; Fig. 6B), coupled with reduced activities of antioxidant enzymes including SOD (F (Al-Wadei et al., 2009; Wang et al., 2019) = 52.06, *p* < 0.001; Fig. 6C), CAT (F (Al-Wadei et al., 2009; Wang et al., 2019) = 20.32, *p* < 0.001; Fig. 6D), and TT (F (Al-Wadei et al., 2009; Wang et al., 2019) = 14.37, *p* < 0.001; Fig. 6E) compared to the vehicle group. Administration NG at 50 mg/kg during nicotine exposure significantly reduced MDA and Nit levels and enhanced SOD and CAT activity (*p* < 0.05; Fig. 6A–D). Treatment with 100 mg/kg NG during nicotine exposure and withdrawal produced more pronounced antioxidant effects, including substantial reductions in MDA (*p* < 0.01 and *p* < 0.001; Fig. 6A) and Nit (*p* < 0.01 and *p* < 0.001; Fig. 6B), and enhanced SOD, CAT, and TT activities (*p* < 0.01 and *p* < 0.001; Fig. 6C–E) compared to the nicotine group. NG alone did not alter oxidative stress markers or antioxidant enzyme activity compared to the vehicle (Fig. 6A–E).

### 3.5 NG ameliorates neuroinflammation and Neurodegenerative Markers

**Fig. 7:**
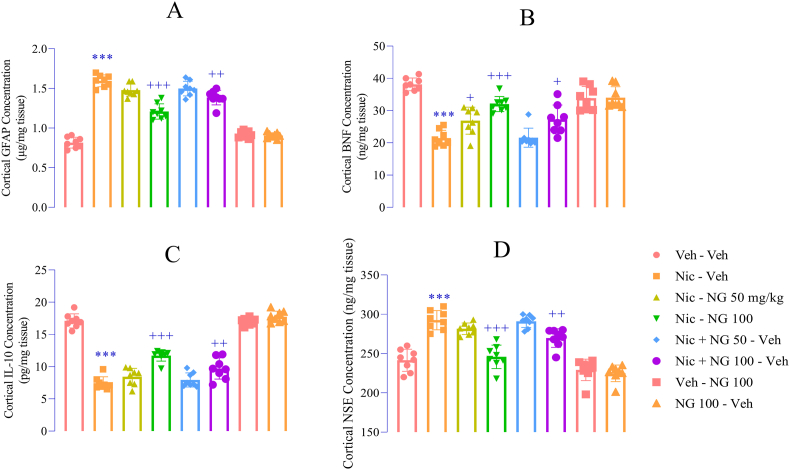
*Effects of Naringenin on Neuroinflammatory and Neurodegenerative Markers* (A) GFAP levels; (B) BDNF levels; (C) IL-10 levels; (D) NSE levels. Data are expressed as mean ± SEM (n = 8). Significant differences: *p* < 0.05, ∗*p* < 0.01, ∗∗*p* < 0.001 vs. vehicle group; +*p* < 0.05, ++*p* < 0.01, +++*p* < 0.001 vs. nicotine group.

Nicotine withdrawal triggered significant neuroinflammatory and neurodegenerative changes. There was a robust increase in GFAP expression (F (Al-Wadei et al., 2009; Wang et al., 2019) = 133.7, *p* < 0.001; Fig. 7A), accompanied by a reduction in neurotrophic BDNF levels (F (Al-Wadei et al., 2009; Wang et al., 2019) = 27.65, *p* < 0.001; Fig. 7B), a decrease in the anti-inflammatory cytokine IL-10 (F (Al-Wadei et al., 2009; Wang et al., 2019) = 132.5, *p* < 0.001; Fig. 7C), and an elevation in NSE (F (Al-Wadei et al., 2009; Wang et al., 2019) = 43.10, *p* < 0.001; Fig. 7D) compared to the vehicle group. Notably, treatment with NG at 100 mg/kg during both the nicotine exposure and withdrawal phases significantly attenuated GFAP expression (*p* < 0.01 and *p* < 0.001; Fig. 7A), restored BDNF (*p* < 0.05 and *p* < 0.001; Fig. 7B) and IL-10 levels (*p* < 0.01 and *p* < 0.001; Fig. 7C), and reduced NSE levels (*p* < 0.01 and *p* < 0.001; Fig. 7D), compared to nicotine group. Importantly, NG administration alone did not produce any significant changes in these parameters compared to the vehicle group (Fig. 6A–E).

## 4 Discussion

Although the behavioral and neurochemical consequences of nicotine dependence and withdrawal are well-documented in both humans and animal models (Haidary et al., 2024; Hossaini et al., 2025c), nicotine use remains alarmingly prevalent among adolescents (Beckmann et al., 2024). The findings of the current study further substantiate this concern by demonstrating that nicotine withdrawal in adolescent rats induces significant neurobehavioral and biochemical alterations, indicative of withdrawal-associated neuropathology. Behavioral analyses revealed pronounced anxiety-like responses. In the OFT, nicotine-withdrawn rats spent significantly less time in the central zone and more time at the periphery, reflecting anxiety-induced avoidance of exposed areas (Amiry et al., 2023; Cohen et al., 2015). This anxiogenic profile was corroborated by performance in the EPM, where rats exhibited reduced time in the open arms and increased preference for the closed arms—hallmarks of elevated anxiety under stress-inducing conditions (Amiry et al., 2023). These findings underscore the vulnerability of adolescent rats to withdrawal-induced emotional dysregulation and are consistent with existing literature on nicotine withdrawal syndromes (Hossaini et al., 2025a; Amiry et al., 2023; Haidary et al., 2024). Importantly, administration of NG significantly ameliorated these anxiety-like behaviors in a dose- and time-dependent manner. Administration of NG at 100 mg/kg during both the nicotine exposure and withdrawal phases effectively reversed withdrawal-induced behavioral impairments, as evidenced by restored central zone activity in the OFT and increased open-arm exploration in the EPM. Notably, even a lower NG dose (50 mg/kg), when administered during the withdrawal phase, was sufficient to attenuate anxiety-like responses, indicating an anxiolytic effect. The absence of behavioral changes in rats treated with NG alone (100 mg/kg, without nicotine exposure) underscores its lack of inherent anxiogenic or anxiolytic effects under baseline, non-stress conditions.

Nicotine withdrawal is associated not only with anxiety-like behaviors but also with depressive-like states. The FST is a widely validated model for assessing behavioral despair and coping strategies, wherein increased immobility and reduced active behaviors (struggling and swimming) are interpreted as indicators of depression-like phenotypes (Kraeuter et al., 2019b; Yankelevitch-Yahav et al., 2015). In the present study, rats undergoing nicotine withdrawal exhibited a significant increase in immobility duration, alongside marked reductions in both struggling and swimming times. These behavioral alterations are indicative of a withdrawal-induced depressive state and align with clinical observations of affective dysregulation during smoking cessation (Kraeuter et al., 2019b). Crucially, administration of NG attenuated these depressive-like behaviors in a dose- and phase-dependent manner. Treatment with NG at 50 mg/kg during the withdrawal phase significantly reduced immobility time, suggesting partial restoration of active coping behavior. More prominently, NG administered at 100 mg/kg across both the nicotine exposure and withdrawal periods elicited robust antidepressant-like effects. This higher dose significantly increased both struggling and swimming durations while concurrently decreasing immobility, reflecting a shift from passive despair to active problem-solving behaviors. Interestingly, NG administered alone (i.e., in non-withdrawn vehicle rats) also resulted in reduced immobility and enhanced swimming behavior, suggesting a mild intrinsic antidepressant-like effect even under baseline conditions.

Epidemiological evidence has linked higher dietary intake of flavonoids, including naringenin, to a reduced incidence of anxiety-related disorders, likely due to their capacity to regulate key neurotransmitters involved in mood and affective processing, such as serotonin and dopamine (Jin et al., 2025; Nachammai et al., 2021). NG's neuroprotective effects may also contribute to its influence on anxiety. It has been shown to enhance the survival of dopaminergic neurons and support neurotrophic factors, which can help stabilize mood and reduce anxiety symptoms (Wang et al., 2019). These neurobiological actions are especially relevant in the context of adolescence, a critical developmental window characterized by heightened neuroplasticity and vulnerability to substance-induced neurobehavioral disturbances (Holliday et al., 2016a; Slawecki et al., 2004). The capacity of NG to attenuate anxiety-like behaviors in adolescent subjects highlights its potential as a novel therapeutic strategy for nicotine dependence, particularly in facilitating cessation efforts and minimizing relapse risk driven by withdrawal-associated affective disturbances.

The PFC plays a central role in the regulation of emotional and cognitive processes, including the modulation of anxiety and depression through its influence on executive functioning and affective responses (Kenwood et al., 2022; Fitzgerald et al., 2019). Dysregulated PFC activity has been consistently implicated in the pathophysiology of anxiety disorders and major depressive disorder (Shiba et al., 2016). Notably, the serotonergic and dopaminergic systems within the PFC are highly susceptible to disruption during nicotine withdrawal, contributing to affective instability and maladaptive coping behaviors (Albert et al., 2014; Arzoo and Ramesh, 2025). This neurotransmitter imbalance is particularly relevant in the context of nicotine dependence, where the dynamic interplay between dopamine and serotonin pathways significantly influences the severity of withdrawal symptoms and the likelihood of relapse (Avelar and George, 2022). In the present study, nicotine withdrawal led to marked reductions in dopamine and serotonin levels in the PFC, accompanied by decreased concentrations of their respective metabolites, DOPAC and 5-HIAA. These findings indicate impaired monoaminergic turnover (Picciotto et al., 2002), which likely underlies the withdrawal-induced emotional blunting, anhedonia, and anxiety observed behaviorally. Importantly, NG treatment effectively normalized these neurochemical disruptions in a dose- and phase-dependent manner. Administration of NG at 100 mg/kg during both nicotine exposure and withdrawal phases significantly restored dopamine and DOPAC levels, suggesting enhanced dopaminergic function—potentially via increased synthesis, reduced catabolism, or improved neuronal resilience. Similarly, NG treatment elevated serotonin and 5-HIAA concentrations, implying beneficial modulation of serotonergic tone. One particularly notable finding was the nicotine withdrawal-induced upregulation of MAO-A activity. This enzymatic elevation contributes to the accelerated degradation of dopamine and serotonin, thereby compounding monoaminergic deficits. In addition, heightened MAO-A activity increases oxidative stress through hydrogen peroxide generation during amine catabolism, further exacerbating neurochemical instability (Kaludercic et al., 2010; Nocito et al., 2007). Collectively, these data suggest that NG not only preserves monoamine availability but may also exert neuroprotective effects by mitigating MAO-A, thereby offering a mechanism for restoring emotional homeostasis during nicotine withdrawal. NG's capacity to significantly inhibit MAO-A activity suggests a dual mechanism of action: preservation of monoamine neurotransmitter levels and attenuation of oxidative stress.

Importantly, NG administered in isolation (i.e., in nicotine-naïve animals) did not produce significant alterations in dopamine, serotonin, their primary metabolites (DOPAC and 5-HIAA), or MAO-A activity. This indicates that NG does not function as a psychostimulant nor disrupt normative neurotransmitter dynamics under physiological conditions. Rather, its effects appear to be selectively neurorestorative, emerging primarily in response to withdrawal-induced neurochemical deficits. These findings collectively support the hypothesis that NG acts as a context-dependent neuromodulator capable of restoring monoaminergic homeostasis during nicotine withdrawal. By simultaneously enhancing dopamine and serotonin availability and suppressing their enzymatic degradation through MAO-A inhibition, NG may alleviate both the affective and cognitive components of withdrawal.

Nicotine withdrawal has been consistently linked to the induction of oxidative stress within the PFC, a region critically involved in mood regulation and executive function (Amiry et al., 2023; Haidary et al., 2024). This oxidative imbalance is strongly associated with the emergence of behavioral symptoms such as anxiety and depression during withdrawal periods. For example, prior research has demonstrated that nicotine withdrawal significantly elevates oxidative stress markers in the brain, correlating with both cognitive deficits and emotional dysregulation (Hossaini et al., 2025a; Amiry et al., 2023). In the present study, nicotine withdrawal profoundly disrupted oxidative balance in the PFC, as evidenced by increased levels of MDA and Nit, alongside a concurrent decrease in endogenous antioxidant defenses, including SOD, CAT, and TT. These findings align with previous reports indicating that the abrupt cessation of chronic nicotine exposure triggers a surge in reactive oxygen species (ROS) and reactive nitrogen species (RNS), which contribute to neuronal dysfunction and affective disturbances (Zhang et al., 2020; Naik et al., 2014; Shaik et al., 2021). The elevation in MDA reflects enhanced lipid peroxidation—a destructive process wherein ROS attack polyunsaturated fatty acids in neuronal membranes—leading to membrane destabilization and cellular damage (Tirani and Haghjou, 2019; Moazamian et al., 2015). Similarly, increased Nit levels are indicative of elevated nitrosative stress, which, in conjunction with oxidative stress, can impair mitochondrial integrity, disrupt cellular respiration, and initiate neuroinflammatory cascades (Tripodi et al., 2025; Mastrocola et al., 2005).

The observed reductions in SOD, CAT, and TT further suggest a significant depletion of the brain's intrinsic antioxidant defense systems during nicotine withdrawal. This loss of redox buffering capacity renders neurons particularly susceptible to oxidative damage, thereby exacerbating the neurobiological underpinnings of withdrawal-induced emotional and cognitive impairments (Salminen and Paul, 2014; Walker et al., 2014). Importantly, administration of NG effectively reversed nicotine withdrawal–induced oxidative disruptions in a dose-dependent manner. Treatment with NG at 50 mg/kg during the exposure phase was sufficient to significantly reduce MDA and Nit levels, while simultaneously enhancing the activity of key antioxidant enzymes, SOD and CAT—indicating moderate yet meaningful antioxidant effects. Notably, the most robust benefits were observed with the higher dose of NG (100 mg/kg) administered across both the nicotine exposure and withdrawal periods. This regimen not only produced a significant reduction in oxidative stress biomarkers (MDA and Nit) but also markedly elevated all three antioxidant defense indicators—SOD, CAT, and TT. These findings suggest that NG confers both preventative and restorative protection against nicotine withdrawal–associated oxidative stress. Its ability to suppress lipid peroxidation and nitrosative burden, while simultaneously replenishing endogenous antioxidant capacity, highlights its potential as a neuroprotective agent capable of stabilizing redox homeostasis during withdrawal. Such multi-faceted antioxidant action may be critical in mitigating the neurobiological and behavioral consequences of nicotine cessation.

The antioxidant properties of NG appear to arise from multiple, complementary mechanisms. First, NG likely functions as a direct free radical scavenger, neutralizing reactive oxygen and nitrogen species (ROS/RNS) to prevent cellular damage. In parallel, NG may enhance endogenous antioxidant defenses by activating key transcriptional regulators, most notably nuclear factor erythroid 2–related factor 2 (Nrf2). Nrf2 governs the expression of antioxidant response element (ARE)-driven genes, including those encoding enzymes such as SOD, CAT, and glutathione-related systems, which are critical for maintaining cellular redox homeostasis (Arafah et al., 2020). In addition to this transcriptional regulation, NG may indirectly mitigate oxidative stress by inhibiting MAO-A—an enzyme that catalyzes the oxidative deamination of monoamines and generates hydrogen peroxide as a byproduct, thereby contributing to ROS accumulation (Ali et al., 2017). This dual mode of action—both direct antioxidant activity and suppression of oxidative byproducts—highlights NG's therapeutic promise in combating oxidative stress–related neuropathology (Hernández-Aquino and Muriel, 2018). The protective effects of NG in the PFC are particularly significant. Oxidative stress in the PFC has been implicated in impairments of cognitive flexibility, emotional regulation, and increased vulnerability to relapse in individuals with substance use disorders (Jones and Graff-Radford, 2021). These concerns are further amplified in adolescents, whose PFC regions are still undergoing critical stages of neurodevelopment and are thus more susceptible to oxidative insults during nicotine withdrawal (Yuan et al., 2015; Chen et al., 2025). Furthermore, the PFC is essential for executive functions, including cognitive flexibility and emotional regulation, which are often compromised in individuals with substance use disorders (Sargin et al., 2019). Therefore, NG's antioxidant effects could play a vital role in safeguarding the developing brain during cessation and recovery from nicotine dependence. Notably, NG administration alone did not alter oxidative stress markers or antioxidant enzyme levels, supporting its safety and specificity. This indicates that NG selectively restores redox balance when oxidative burden is elevated but does not interfere with physiological oxidative signaling under normal conditions.

Nicotine withdrawal is increasingly recognized as a neuroinflammatory state that exacerbates neural dysfunction and heightens susceptibility to relapse—effects that are especially pronounced in adolescents (Mahajan et al., 2021a). The adolescent brain, undergoing critical phases of maturation, is particularly vulnerable to the neuroimmune consequences of nicotine exposure. Emerging evidence indicates that nicotine withdrawal in this age group elicits pronounced neuroinflammation, characterized by the activation of microglial cells—central mediators of the brain's innate immune response (Mahajan et al., 2021b). Microglial activation has been associated with both the behavioral symptoms of addiction and the cognitive impairments observed during abstinence. Moreover, nicotine withdrawal has been shown to disrupt functional brain network connectivity, potentially underpinning the cognitive deficits and increased craving states reported during cessation (Lerman et al., 2014). These neurobiological disturbances can severely impair emotional regulation and executive functioning in adolescents, increasing the risk of relapse and complicating recovery efforts (Holliday et al., 2016b). Beyond microglial activation, dysregulation of monoaminergic systems—particularly serotonergic and dopaminergic pathways—also contributes to the pro-inflammatory milieu. Serotonin and dopamine are not only essential for mood and motivation but also play key roles in modulating neuroimmune responses. Perturbations in these neurotransmitter systems have been linked to increased release of pro-inflammatory cytokines and chemokines, further amplifying neuroinflammatory signaling within the central nervous system (Saggu et al., 2025).

In the present study, nicotine withdrawal induced a neurobiological profile consistent with concurrent neuroinflammation and neurodegeneration in the PFC. This was evidenced by significant upregulation of GFAP, a well-established marker of astrocyte reactivity and neuroinflammatory activation (Li et al., 2020). Concurrently, nicotine withdrawal led to reductions in BDNF and IL-10, both of which are critical for maintaining neuronal health and anti-inflammatory balance. Elevated levels of NSE, a marker of neuronal injury, further confirmed the presence of neurodegenerative processes (Haque et al., 2018). Collectively, these findings indicate that nicotine withdrawal elicits a pathological state marked by glial activation, impaired neurotrophic support, reduced anti-inflammatory signaling, and neuronal damage—highlighting the converging roles of inflammation and degeneration in the prefrontal cortex during abstinence.

The observed increase in GFAP expression during nicotine withdrawal indicates heightened astrocytic reactivity, a hallmark of neuroinflammatory responses. Activated astrocytes are known to secrete pro-inflammatory cytokines and exacerbate oxidative stress, thereby contributing to neuronal dysfunction and synaptic instability (Namba et al., 2020). Concurrently, a significant reduction in BDNF—a key neurotrophin that supports synaptic plasticity, neurogenesis, and neuronal survival—suggests a neurobiological environment unfavorable for neuronal maintenance and repair (Colucci-D’Amato et al., 2020).

The simultaneous decline in IL-10, a potent anti-inflammatory cytokine, further reflects an imbalance in immune homeostasis, tipping the neuroimmune response toward a pro-inflammatory state (Iyer and Cheng, 2012). This dysregulation exacerbates the neurotoxic milieu, likely fueling a feedback loop of neuroinflammation and oxidative stress. Elevated NSE levels further reinforce the presence of neuronal stress or injury—likely driven by a combination of glial activation, excitotoxicity, and oxidative damage (Haque et al., 2018). As a reliable biomarker of neuronal injury, increased NSE levels suggest that withdrawal initiates widespread neurodegenerative processes in the PFC (Hong et al., 2020).

Remarkably, administration of NG at 100 mg/kg throughout both the nicotine exposure and withdrawal phases significantly reversed these pathological alterations. NG markedly reduced GFAP expression, indicating a suppression of astrocytic activation and attenuation of the neuroinflammatory cascade. Restoration of BDNF levels by NG suggests enhanced neurotrophic support, which may underlie the observed improvements in cognitive and affective behaviors by promoting neuronal resilience, synaptic integrity, and repair mechanisms. Additionally, NG elevated IL-10 concentrations during the withdrawal phase, suggesting a shift toward an anti-inflammatory profile. NG treatment also led to a significant reduction in NSE levels, indicating mitigation of neuronal injury and preservation of cellular integrity. The absence of any significant changes in these inflammatory and degenerative markers in non-withdrawn rats treated with NG underscores its safety and selective efficacy in pathologically altered states. This context-dependent action enhances its therapeutic appeal, as it suggests NG restores homeostasis without interfering with normal physiological processes. The multifaceted effects of NG observed in this study suggest a complex mechanism of action involving the suppression of neuroinflammation—potentially through downregulation of nuclear factor kappa-light-chain-enhancer of activated B cells (NF-Κb) signaling pathways (Dou et al., 2013; Shi et al., 2009)—attenuation of oxidative stress via upregulation of endogenous antioxidant systems (Arafah et al., 2020), and promotes neuronal survival via BDNF-mediated pathways (Saati, 2025).

It is important to acknowledge some limitations inherent to this study. A notable limitation of the present study is the absence of female rats. Sex differences have been consistently reported in both humans and animal models, with females often exhibiting distinct behavioral, neurochemical, and affective responses to nicotine exposure and withdrawal (Becker and Koob, 2016). Fluctuations in ovarian hormones, particularly estrogen and progesterone, modulate nicotine reinforcement, withdrawal severity, and susceptibility to relapse (Becker and Koob, 2016). Excluding females due to hormonal variability, while common, limits the translational relevance of preclinical findings (Shansky and Woolley, 2016). Incorporating female subjects in future studies is essential to capture sex-specific neurobehavioral and neurochemical outcomes and to develop interventions, such as NG, that are effective across both sexes.

## 5 Conclusion

This study provides evidence that nicotine withdrawal during adolescence leads to profound neurobehavioral impairments, including anxiety- and depression-like behaviors, as well as significant disruptions in dopaminergic and serotonergic neurotransmission, oxidative homeostasis, and neuroinflammatory signaling within the PFC. Importantly, treatment with NG administered across both exposure and withdrawal phases—robustly mitigated these detrimental effects. NG restored monoaminergic balance, inhibited MAO-A activity, reduced oxidative and nitrosative stress, enhanced endogenous antioxidant defenses, and attenuated markers of neuroinflammation and neuronal injury. These findings highlight NG's multifaceted neuroprotective actions and suggest its potential as a safe and effective therapeutic agent for alleviating the neuropsychiatric consequences of nicotine withdrawal, especially in adolescents. Future studies are warranted to further explore its mechanisms of action and translational potential in clinical settings.

## Credit author statement

Murtaza Haidary: Conceptualization, Supervision, Project administration, Formal Analysis, Writing – Review & Editing (Corresponding Author).

Yahya Samadi, Zakaria Rezai, Atiqullah Sadaqat, Mohammad Ali Ahmadi, Jamshid Gholami, Mohammad Mahdi Mohammadi: Investigation, Data Curation, Writing – Original Draft. Mohammad Taqi Shojae: Writing – review and editing.

## Funding

This research received no specific grant from any funding agency in the public, commercial, or not-for-profit sectors.

## Declaration of competing interest

The authors declare that there are no conflicts of interest, financial or otherwise, associated with this study. All research was conducted independently and without any commercial or financial relationships that could be construed as potential conflicts of interest.

## Data Availability

The datasets generated and analyzed during the current study are available from the corresponding author upon reasonable request.
