# Supplementary material for: Neuroprotective effects of naringenin on nicotine-induced anxiety and depression: Involvement of monoaminergic systems, oxidative stress, and neuroinflammation on male rats
Source: Curr Res Pharmacol Drug Discov. 2025 Dec 4;10:100242. doi: 10.1016/j.crphar.2025.100242 (PMC12750789; doi:10.1016/j.crphar.2025.100242)
Supplement: Multimedia component 1 [file mmc1.pdf]

Authorship change request

Important information. Please read before completing this form.
This form is to request any change in authorship (additions, removals, or reordering) after the submission of a manuscript, including changes in corresponding authors, if any. This form should not be used for changes requested *after* publication or for name changes or corrections.

Prior to completing this form, all authors should carefully review the 'Duties of Authors' section of the Elsevier publishing ethics policy, and in particular, the sections on:

- Authorship of the paper
- The use of generative AI and AI-assisted technologies in scientific writing and in figures, images and artwork

Please also carefully review the journal's guide for authors (this might also be referred to as 'instructions for authors') as some journals may have additional authorship criteria (e.g., the ICMJE guidelines for authorship).

The publisher and editor cannot investigate or mediate any authorship disputes. If you are unable to obtain agreement from all authors, including those you intend to remove, we recommend seeking guidance from your institution. We will not consider your change request and will not proceed with the publication of your manuscript until all outstanding authorship disputes are resolved.

If your manuscript is still under consideration, this completed form should be submitted in Editorial Manager for consideration as part of your revision submission (use the 'cover letter' file type).

If your manuscript has already been accepted and is in the proofing or production stages, please return this completed form to the Journal Manager.

If the final version of your manuscript has already been published, a corrigendum will be required. Please see the Article Correction, Retraction and Removal Policy.

Section 1. Submission information

To be completed by the corresponding author.

**Submission information**	
**Journal title**	Current Research in Pharmacology and Drug Discovery
**Manuscript number and/or article number**	CRPHAR-D-25-00069
**Manuscript title**	Neuroprotective Effects of Naringenin on Nicotine-Induced Anxiety and Depression: Involvement of Monoaminergic Systems, Oxidative Stress, and Neuroinflammation

**Change(s) requested** (indicate as appropriate)	
☑ Add new author(s)	☐ Remove author(s)
☐ Change the corresponding author	☐ Change the order of authors

Section 2. Author(s) added or removed

Complete one table for each author to be added or removed. Please include as much detail as possible in the "Reason for change" section so that we can evaluate if the change is necessary. At a minimum, this should include an explanation for why the change is being requested and why the author was/was not included in the original author list.

If the form is not provided, incomplete, or the reasons provided are insufficiently detailed or do not address the points above, your request will be denied and your submission may be rejected.

Unless instructed by the editor, further changes to the author list without an approved authorship change request will result in the rejection of your submission, or retraction, if the article has already been published.

2.1 Author information

**Given/first name(s)**	Mohammad Taqi
**Family/last name**	Shojae
**Email address**	m.taqishojaie2018@gmail.com
**Institution**	Khatam Al- Nabieen University, Kabul, Afghanistan
**Change(s) requested** (indicate as appropriate)	☑ Add new author ☐ Remove author ☐ Make the corresponding author

Individual contributions per CRediT Contributor Roles Taxonomy (required for author additions only)

☐ Conceptualization	☐ Data curation	☐ Formal analysis
☐ Funding acquisition	☐ Investigation	☐ Methodology
☐ Project administration	☐ Resources	☐ Software
☐ Supervision	☐ Validation	☐ Visualization
☐ Writing – original draft	☑ Writing – review & editing	

Reason for the change

Mr. Mohammad Taqi Shojae was added to the author list because he made substantial intellectual contributions during the revision process, including critical review of the content, improving the clarity and structure of the manuscript, and contributing to the interpretation of the findings. His involvement meets the journal's criteria for authorship.

2.2 Author information

**Given/first name(s)**	
**Family/last name**	
**Email address**	
**Institution**	
**Change(s) requested** (indicate as appropriate)	☐ Add new author ☐ Remove author ☐ Make the corresponding author

Individual contributions per CRediT Contributor Roles Taxonomy (required for author additions only)

☐ Conceptualization	☐ Data curation	☐ Formal analysis
☐ Funding acquisition	☐ Investigation	☐ Methodology
☐ Project administration	☐ Resources	☐ Software
☐ Supervision	☐ Validation	☐ Visualization
☐ Writing – original draft	☐ Writing – review & editing	

Reason for the change

2.3 Author information

**Given/first name(s)**	
**Family/last name**	
**Email address**	
**Institution**	
**Change(s) requested** (indicate as appropriate)	☐ Add new author ☐ Remove author ☐ Make the corresponding author

Individual contributions per CRediT Contributor Roles Taxonomy (required for author additions only)

☐ Conceptualization	☐ Data curation	☐ Formal analysis
☐ Funding acquisition	☐ Investigation	☐ Methodology
☐ Project administration	☐ Resources	☐ Software
☐ Supervision	☐ Validation	☐ Visualization
☐ Writing – original draft	☐ Writing – review & editing	

Reason for the change

2.4 Author information

**Given/first name(s)**	
**Family/last name**	
**Email address**	
**Institution**	
**Change(s) requested** (indicate as appropriate)	☐ Add new author ☐ Remove author ☐ Make the corresponding author

Individual contributions per CRediT Contributor Roles Taxonomy (required for author additions only)

☐ Conceptualization	☐ Data curation	☐ Formal analysis
☐ Funding acquisition	☐ Investigation	☐ Methodology
☐ Project administration	☐ Resources	☐ Software
☐ Supervision	☐ Validation	☐ Visualization
☐ Writing – original draft	☐ Writing – review & editing	

Reason for the change

2.5 Author information

**Given/first name(s)**	
**Family/last name**	
**Email address**	
**Institution**	
**Change(s) requested** (indicate as appropriate)	☐ Add new author ☐ Remove author ☐ Make the corresponding author

Individual contributions per CRediT Contributor Roles Taxonomy (required for author additions only)

☐ Conceptualization	☐ Data curation	☐ Formal analysis
☐ Funding acquisition	☐ Investigation	☐ Methodology
☐ Project administration	☐ Resources	☐ Software
☐ Supervision	☐ Validation	☐ Visualization
☐ Writing – original draft	☐ Writing – review & editing	

Reason for the change

*Add additional page(s) as needed for more requested changes.

Section 3. Author order and agreement

Provide the author list in the order that you would like it to be published.

The form must be signed individually by each author, including any added/removed authors. In cases of consortia group authorship, the corresponding author may sign on behalf of the group.

While handwritten signatures are acceptable, we highly encourage the use of electronic signature software (DocuSign, Adobe Sign, HelloSign, or similar) with valid e-signatures. These signatures should reflect your institutional information and email, as provided in the author list below. **Typed signatures or images of signatures will not be accepted**.

By signing this form all authors agree:

1) that they have read and acknowledge the publishing ethics policies linked in the "Important Information" section of this form;
2) agree to the addition and/or removal of the authors listed in section 2 and to the revised order of the author list in this section 3, and;
3) that all information provided accurately reflects the authorship of the article.

Agreement of removed author(s)

Full name	Email address	Signature	Date

*Add additional page(s) as needed.

Proposed author list

Order	Full name	Email address	Signature	Date
01	Murtaza Haidary	sadramurtaza@gmail.com		15-10-2025
02	Yahya Samadi	yahyasamadi313@gmail.com		15-10-2025
03	Zakaria Rezai	zakariarezai4989@gmail.com		15-1.-2.25
04	Atiqullah Sadaqat	atiqullahsadaqat61@gmail.com		15-1.-2025
05	Mohammad Ali Ahmadi	muhammadahmady87@gmail.com	Ali	15-10-2025
06	Jamshid Gholami	jamshidgholami0101@gmail.com	Gholami	15-10-2025
07	Mohammad Mahdi Mohammadi	mohammadmahdimohammadi46@gmail.com	mohammadi	15-10-2025
08	Mohammad Taqi Shojae	m.taqishojaie2018@gmail.com	Taqi shojae	15-10-2025
09				
10				
11				
12				
13				
14				
15				
16				
17				
18				
19				
20				
21				
22				
23				
24				
25				

*Add additional page(s) as needed.
